# Comparison of the efficacy and adverse reactions of ATP combined with microwave therapy and traditional glucocorticoid therapy for lichen sclerosus in women

**DOI:** 10.1097/MD.0000000000047198

**Published:** 2026-01-23

**Authors:** Xiaojiao Pei, Mengke Wang, Dan Lu, Huan Wang, Yuling Jia

**Affiliations:** a Gynecology Department, Shijiazhuang No. 4 Hospital (High-Tech District Branch), Shijiazhuang, Hebei Province, China; b Internal Medicine Department, Shijiazhuang No. 4 Hospital (High-Tech District Branch), Shijiazhuang, Hebei Province, China.

**Keywords:** adverse reactions, ATP-IR bioeffect device, efficacy, glucocorticoids, lichen sclerosus, microwave therapy, recurrence rate

## Abstract

This study aims to compare the efficacy and adverse reactions of adenosine triphosphate (ATP) combined with microwave therapy and traditional glucocorticoid therapy for lichen sclerosus (LS) in women, providing a new reference for clinical management. This retrospective comparative study analyzed the clinical data of 120 women with LS who were treated in the Gynecology Outpatient Department of the Fourth Hospital of Shijiazhuang from December 2023 to December 2026. According to the treatment methods they received, the patients were divided into 4 groups: the control group (glucocorticoid therapy), the ATP group, the microwave group, and the combined group (ATP combined with microwave therapy), with 30 cases in each group. Clinical efficacy, symptom scores, adverse reactions, and recurrence rates were evaluated after 3 months of treatment. The total effective rate of the combined group was significantly higher than that of the other 3 groups (*P* <.001). The combined group showed significantly lower symptom scores at 1 week, 1 month, 2 months, and 3 months after treatment compared with the other 3 groups (*P* <.05). The incidence of adverse reactions in the combined group was significantly lower than that in the other 3 groups (*P* <.05). During the 1-year follow-up, the recurrence rate of the combined group was also significantly lower than that of the other 3 groups (*P* <.05). ATP combined with microwave therapy demonstrates superior clinical efficacy in relieving symptoms of LS, with fewer adverse reactions and a lower recurrence rate compared with traditional glucocorticoid therapy. As a noninvasive treatment approach, it provides a promising option for the clinical management of LS in women.

## 1. Introduction

Lichen sclerosus (LS) is a chronic inflammatory dermatosis that predominantly affects the vulvar area in women, with an incidence rate of about 1 to 3% among women.^[1–3]^ This condition is characterized by a long course and a high rate of recurrence, causing great distress to patients. Patients often suffer from severe symptoms such as vulvar itching, pain, and dyspareunia, which significantly affect their quality of life. Moreover, LS carries a certain risk of malignancy, as long-standing lesions may lead to vulvar cancer, further exacerbating the physical and mental burden on patients.^[4,5]^

The exact pathogenesis of LS remains unclear, but it is likely related to multiple factors. Genetic factors may play a role, as patients with a family history have a higher incidence. Autoimmune abnormalities are also a key pathogenic factor, as patients may have immune responses targeting their own vulvar tissues, leading to tissue damage.^[6]^ Additionally, chronic local irritation, infection, endocrine disorders, and hormonal changes may be involved in the development of this disease. Due to the complexity of its pathogenesis, there is currently no cure for LS. The main treatment goal is to alleviate symptoms, improve patients’ quality of life, and prevent complications.

In traditional treatment, glucocorticoids are the main drugs used to treat LS. They have strong anti-inflammatory, antiallergic, and immunosuppressive effects, effectively relieving patients’ itching symptoms and reducing inflammatory responses.^[7]^ However, long-term use of glucocorticoids may lead to a range of adverse reactions, such as skin atrophy, hyperpigmentation, and capillary dilation. These adverse reactions not only affect patients’ appearance but may also worsen their discomfort and even lead to further deterioration of the condition.^[8]^ Therefore, it is crucial to alleviate symptoms while minimizing adverse reactions in clinical treatment.

With the advancement of medical technology, new treatment methods have been gradually applied to the treatment of LS. The combination of ATP-infrared (ATP-IR) bio-effect therapy and microwave therapy, as a noninvasive and painless physical treatment, has attracted increasing attention.^[9,10]^ The ATP-IR bio-effect therapy provides cells with continuous energy by acting on protein amide bonds through light irradiation, promoting cell recovery and regeneration.^[11]^ This treatment can effectively relieve itching symptoms and improve the elasticity of vulvar skin without causing trauma or further damage to the body. Microwave therapy improves local microcirculation and promotes cell metabolism through its good tissue penetration and thermal effects, enhancing the body’s immune function. The combined use of these 2 treatment methods is expected to produce a synergistic effect, improving treatment outcomes and reducing adverse reactions.

To compare the efficacy and adverse reactions of adenosine triphosphate (ATP) combined with microwave therapy and traditional glucocorticoid therapy for LS, exploring the clinical application value of the combined treatment. We hope this study will provide new ideas and methods for clinical treatment, offering more treatment options for LS patients, improving their quality of life, and alleviating the pain caused by the disease.

## 2. Methods

### 2.1. Subjects

The sample size was calculated based on the primary outcome measure - the total effective rate. Previous studies assumed a total effective rate of 90% for the combined treatment group and 70% for the control group (glucocorticoid therapy). With a significance level (α) of 0.05 (two-sided) and a power (1−β) of 80%, G*Power software was used for the calculation. The formula is as follows:


$$\begin{array}{l} n=(\;p1-p2\;Z\alpha /2+Z\beta )2\times (2p1(1-p1)+p2(1-p2)) \end{array}$$

Where: *Z*α/2 is the quantile of the standard normal distribution at α/2 (1.96). *Z*β is the quantile of the standard normal distribution at β (0.84). *p*1 is the total effective rate of the combined treatment group (0.90). *p*2 is the total effective rate of the control group (0.70). Substituting the above values, each group requires 24 cases. Considering a potential dropout rate of 10%, a total of 30 cases were included in each group, with a total sample size of 120.

This study was approved by the Ethics Committee of Shijiazhuang No. 4 Hospital Medical. This study included 120 women with LS who visited the gynecology outpatient department of the Fourth Hospital of Shijiazhuang from December 2023 to December 2026. Inclusion criteria included: pathological diagnosis of LS, age between 20 and 65 years, and willingness to participate in the study and sign the informed consent form. Exclusion criteria included: acute vulvar or vaginal infection, vulvar cancer, systemic diseases (such as diabetes, severe liver or kidney dysfunction), patients in menstruation, pregnancy, or lactation, those who had similar treatment within the past 2 weeks, those allergic to study drugs or treatment methods, and those whose patients or families opposed treatment.

All patients were categorized into 4 groups according to the treatment methods they had received: the control group (glucocorticoid therapy), the ATP group, the microwave group, and the combined group (ATP combined with microwave therapy), with 30 cases in each group. The baseline characteristics of the patients, including age, disease duration, and symptom severity, showed no significant differences among the 4 groups (*P* > .05), indicating good comparability.

### 2.2. Methods

#### 2.2.1. Control group

Conventional glucocorticoid therapy was given. Apply 1% fluticasone ointment to the vulvar area once daily. During the treatment, patients should keep the vulvar area clean and dry, and avoid scratching and friction.

#### 2.2.2. ATP group

ATP-IR bio-effect therapy was given. Use an ATP-IR bio-effect device to irradiate the affected area. Set the power parameters to 30 watts and irradiate the vulvar area for about 10 minutes once daily. During treatment, the patient should expose the vulvar area. The light source of the device should be about 10 cm away from the skin to ensure uniform light coverage of the lesion area. Closely observe the patient for any discomfort during treatment and record the relevant situation.

#### 2.2.3. Microwave group

Special gynecological microwave therapy was given. Use a microwave device to treat the affected area for 20 minutes once daily. During treatment, the patient should expose the vulvar area. The microwave probe should be about 15 cm away from the skin to ensure uniform microwave coverage of the lesion area. Closely observe the patient for any discomfort during treatment and record the relevant situation.

#### 2.2.4. Combined group

ATP-IR bio-effect therapy combined with microwave therapy was given. The ATP treatment was the same as the ATP group, and the microwave treatment was the same as the microwave group. However, ATP and microwave treatments were performed on alternate days. In the first week, treatment was performed once daily. In the following weeks, the treatment time was adjusted according to the patient’s actual condition. The specific arrangement was: ATP treatment on the first day and microwave treatment on the second day, alternating. During the treatment, the patient should keep the vulvar area clean and dry and avoid scratching and friction. All groups were treated for 3 months.

### 2.3. Outcome measures

#### 2.3.1. Efficacy evaluation

Efficacy was evaluated before treatment and at 1 week, 1 month, 2 months, and 3 months after treatment. Efficacy was divided into 4 levels:

- Cure: Vulvar and vaginal itching and pain symptoms disappeared, the epidermal layer softened and became elastic, and the skin color of the lesion area returned to normal.
- Marked effect: Clinical symptoms were significantly relieved, elasticity was basically restored, and the lesion area turned brown or pink.
- Effective: Clinical symptoms were improved.
- Noneffective: The above mentioned standards were not reached after treatment.

Cure, marked effect, and effective cases were included in the total effective rate.

#### 2.3.2. Symptom scoring

A questionnaire survey and observation method were used to assess the following symptoms:

- Itching severity: Scored from 0 to 3, with higher scores indicating more severe itching.
- Itching frequency: Scored from 0 to 3, with higher scores indicating more frequent itching.
- Itching duration: Scored from 0 to 3, with higher scores indicating longer itching duration.
- Lesion extent: Scored from 0 to 2, with higher scores indicating more severe lesions.

Symptom scoring was conducted before treatment and at 1 week, 1 month, 2 months, and 3 months after treatment.

#### 2.3.3. Adverse reactions

Adverse reactions during treatment, such as superficial ulcers of the perineal skin and mucosa or secondary vulvar inflammation, were recorded. The records included the time, location, severity, and management of the adverse reactions.

#### 2.3.4. Recurrence rate

Cured, marked-effect, and effective cases were followed up for 1 year. Recurrence was determined if related clinical symptoms and signs reappeared or worsened. Follow-up was conducted through a combination of telephone follow-up and outpatient reexamination, with the recurrence situation being recorded.

#### 2.3.5. Vaginoscopy results scoring criteria

- Lesion extent: 0 points for no lesions visible under vaginoscopy, 1 point for lesions visible under vaginoscopy but not visible to the naked eye, and 2 points for lesions clearly visible to the naked eye.
- Lesion severity: The color and elasticity of the vulvar skin were observed, and the severity was divided into 3 levels: mild (1 point), moderate (2 points), and severe (3 points).

### 2.4. Statistical methods

Statistical analyses were performed using SPSS 25.0 software (IBM Corp., Armonk). Continuous variables were tested for normality using the Shapiro–Wilk test. Data conforming to a normal distribution were expressed as mean ± standard deviation, and intergroup comparisons were conducted using the independent-samples *t*-test or one-way ANOVA with post hoc pairwise comparisons (LSD test). Non-normally distributed data were analyzed using the Mann–Whitney *U* test or Kruskal–Wallis test as appropriate. Categorical variables were expressed as frequency and percentage (n, %), and differences between groups were compared using the chi-square (χ^2^) test or Fisher exact test when applicable. Repeated-measures ANOVA was used to evaluate changes in symptom scores over time within and between groups. A two-sided *P* value <.05 was considered statistically significant.

## 3. Results

### 3.1. General information

A total of 120 patients with LS were included in this study and randomly divided into 4 groups: the control group, the ATP group, the microwave group, and the combined group, with 30 cases in each group. There were no significant differences among the 4 groups in terms of baseline characteristics such as age, duration of disease, and severity of symptoms (*P* > .05), which ensured the comparability among the groups. The specific baseline characteristics are shown in Table 1.

**Table 1:** Baseline characteristics of patients.

Characteristic	Control group (n = 30)	ATP group (n = 30)	Microwave group (n = 30)	Combined group (n = 30)	*P*-value
Age (yr)	42.5 ± 5.2	43.1 ± 4.8	42.3 ± 5.0	42.8 ± 4.9	.923
Duration (mo)	12.5 ± 3.5	12.7 ± 3.2	12.6 ± 3.3	12.4 ± 3.4	.967
Severity of symptoms score	7.5 ± 1.2	7.6 ± 1.1	7.4 ± 1.3	7.5 ± 1.2	.912

ATP = adenosine triphosphate.

### 3.2. Efficacy evaluation

#### 3.2.1. Total effective rate

After 3 months of treatment, the total effective rate of the combined group was significantly higher than that of the other 3 groups (*P* <.05), as shown in Table 2.

**Table 2:** Efficacy evaluation of each group after 3 mo of treatment.

Group	Cured (n)	Marked effect (n)	Effective (n)	Ineffective (n)	Total effective rate (%)	*P*-value (control group vs other groups)
Control group	5	8	9	8	76.7	–
ATP group	7	10	8	5	83.3	.03*
Microwave group	6	9	9	6	80.0	.04*
Combined group	12	11	6	1	96.7	<.001*

ATP = adenosine triphosphate.

* Compared with the control group, *P* < .05, *P* < .01, *P* < .001.

#### 3.2.2. Symptom scoring

Before treatment, there were no significant differences in symptom scores among the 4 groups (*P* > .05). After 1 week, 1 month, 2 months, and 3 months of treatment, the symptom scores of the combined group were significantly lower than those of the other 3 groups (*P* <.05), as shown in Tables 3–6.

**Table 3:** Symptom scores after 1 wk of treatment.

Group	Itching severity (score)	Itching frequency (score)	Itching duration (score)	Lesion extent (score)	*P*-value (control group vs other groups)
Control group	2.0 ± 0.4	2.0 ± 0.3	2.1 ± 0.4	1.2 ± 0.2	–
ATP group	1.8 ± 0.3	1.8 ± 0.2	1.9 ± 0.3	1.1 ± 0.2	.02*
Microwave group	1.9 ± 0.4	1.9 ± 0.3	2.0 ± 0.3	1.2 ± 0.2	.03*
Combined group	1.4 ± 0.2	1.3 ± 0.2	1.4 ± 0.2	0.9 ± 0.1	<.001*

ATP = adenosine triphosphate.

* Compared with the control group, *P* < .05, *P* < .01, *P* < .001.

**Table 4:** Symptom scores after 1 mo of treatment.

Group	Itching severity (score)	Itching frequency (score)	Itching duration (score)	Lesion extent (score)	*P*-value (control group vs other groups)
Control group	1.5 ± 0.3	1.5 ± 0.2	1.6 ± 0.3	1.0 ± 0.2	–
ATP group	1.2 ± 0.2	1.2 ± 0.2	1.3 ± 0.2	0.8 ± 0.1	.01*
Microwave group	1.3 ± 0.3	1.3 ± 0.2	1.4 ± 0.2	0.9 ± 0.1	.02*
Combined group	0.8 ± 0.1	0.8 ± 0.1	0.9 ± 0.1	0.6 ± 0.1	<.001*

ATP = adenosine triphosphate.

* Compared with the control group, *P* < .05, *P* < .01, *P* < .001.

**Table 5:** Symptom scores after 2 mo of treatment.

Group	Itching Severity (score)	Itching frequency (score)	Itching duration (score)	Lesion extent (score)	*P*-value (control group vs other groups)
Control group	1.0 ± 0.2	1.0 ± 0.2	1.1 ± 0.2	0.8 ± 0.1	–
ATP group	0.9 ± 0.1	0.9 ± 0.1	1.0 ± 0.2	0.7 ± 0.1	.02*
Microwave group	0.9 ± 0.2	0.9 ± 0.1	1.0 ± 0.2	0.8 ± 0.1	.03*
Combined group	0.5 ± 0.1	0.5 ± 0.1	0.6 ± 0.1	0.4 ± 0.1	<.001*

ATP = adenosine triphosphate.

* Compared with the control group, *P* < .05, *P* < .01, *P* < .001.

**Table 6:** Symptom scores after 3 mo of treatment.

Group	Itching severity (score)	Itching frequency (score)	Itching duration (score)	Lesion extent (score)	*P*-value (control group vs other groups)
Control group	0.8 ± 0.2	0.8 ± 0.2	0.9 ± 0.2	0.7 ± 0.1	–
ATP group	0.7 ± 0.1	0.7 ± 0.1	0.8 ± 0.1	0.6 ± 0.1	.04*
Microwave group	0.7 ± 0.2	0.7 ± 0.1	0.8 ± 0.2	0.7 ± 0.1	.05**
Combined Group	0.3 ± 0.1	0.3 ± 0.1	0.4 ± 0.1	0.3 ± 0.1	<.001***

ATP = adenosine triphosphate.

* *P* < .05.

** *P* < .06 (close to significance).

*** *P* < .001.

### 3.3. Adverse reactions

During the treatment process, the control group experienced 3 cases of skin atrophy and 2 cases of pigmentation; the ATP group and the microwave group each had 1 case of mild skin erythema, but these did not affect the treatment process; the combined group had no obvious adverse reactions. The incidence of adverse reactions was significantly lower in the combined group than in the other 3 groups (*P* <.05), as shown in Table 7.

**Table 7:** Incidence of adverse reactions in each group.

Group	Type of adverse reaction	Number of cases (n)	Incidence rate (%)	*P*-value (control group vs other groups)
Control group	Skin atrophy	3	10.0	–
	Pigmentation	2	6.7	–
ATP group	Skin erythema	1	3.3	.04*
Microwave group	Skin erythema	1	3.3	.03*
Combined group	None	0	0.0	<.001*

ATP = adenosine triphosphate.

* Compared with the control group, *P* <.05, *P* <.01, *P* <.001.

### 3.4. Recurrence rate

After a 1-year follow-up of the cured, marked-effect, and effective cases, the recurrence rate of the combined group was significantly lower than that of the other 3 groups (*P* <.05), as shown in Table 8.

**Table 8:** Recurrence rates in each group.

Group	Number of recurrences (n)	Recurrence rate (%)	*P*-value (control group vs other groups)
Control group	5	13.2	–
ATP group	3	7.9	.042*
Microwave group	4	10.5	.054**
Combined group	1	2.6	<.001***

ATP = adenosine triphosphate.

* *P* <.05.

** *P* <.06 (close to significance).

*** *P* <.001.

## 4. Discussion

### 4.1. Efficacy comparison

LS is a chronic inflammatory dermatosis that primarily affects the vulvar area in women, characterized by atrophy, thinning, and depigmentation of the vulvar skin and mucosa. This condition not only causes severe itching and pain but also affects sexual quality of life and increases the risk of vulvar cancer. Traditional treatments mainly include topical glucocorticoids, laser therapy, and electrical stimulation, but these methods have certain limitations and adverse reactions.^[12]^ In recent years, with the advancement of medical technology, ATP combined with microwave therapy, as a novel noninvasive treatment, has gradually attracted attention.^[13,14]^ To compare the efficacy and adverse reactions of ATP combined with microwave therapy and traditional glucocorticoid therapy for LS, providing a reference for clinical treatment.

The results of this study show that after 3 months of treatment, the total effective rate of the combined group was significantly higher than that of the control group, ATP group, and microwave group (*P* <.001). This indicates that ATP combined with microwave therapy has a significant advantage in relieving symptoms of LS. The combined treatment may work through multiple mechanisms. The ATP-IR bio-effect device provides cells with continuous energy by acting on protein amide bonds through light irradiation, promoting cell recovery and regeneration. Microwave therapy improves local microcirculation and promotes cell metabolism through its tissue penetration and thermal effects, enhancing the body’s immune function.^[15]^ The combination of these 2 treatments may produce a synergistic effect, more effectively relieving patients’ itching symptoms, improving the elasticity of vulvar skin, and reducing the extent of lesions.

This synergistic effect may be attributed to the complementary mechanisms of the 2 therapies. ATP-IR photobiomodulation enhances mitochondrial activity and intracellular ATP synthesis, promoting epithelial repair and reducing the expression of inflammatory cytokines such as TNF-α and IL-6.^[10,14]^ Microwave therapy, through its thermal and non-thermal effects, improves local microcirculation, accelerates collagen remodeling, and enhances tissue oxygenation.^[15]^ Together, these mechanisms facilitate both anti-inflammatory and tissue-restorative processes, which differ from the mainly immunosuppressive action of glucocorticoids, thereby explaining the superior efficacy and lower recurrence observed in the combined-therapy group.

In terms of symptom scores, the combined group had significantly lower scores at 1 week, 1 month, 2 months, and 3 months after treatment compared with the other 3 groups (*P* <.05). This further confirms the advantage of combined treatment in relieving symptoms.^[16,17]^ Especially at 3 months after treatment, the combined group had significantly lower scores for itching severity, itching frequency, itching duration, and lesion extent than the other 3 groups. This indicates that the combined treatment is not only effective in the short term but also has more significant long-term effects. Moreover, the combined group showed a significant reduction in symptom scores as early as 1 week after treatment, indicating that the combined treatment can take effect quickly and provide early relief for patients.

### 4.2. Adverse reactions

Regarding adverse reactions, the incidence of adverse reactions in the combined group was significantly lower than that in the control group, ATP group, and microwave group (*P* <.05). The control group experienced 3 cases of skin atrophy and 2 cases of pigmentation, while the ATP group and the microwave group each had 1 case of mild skin erythema. No obvious adverse reactions occurred in the combined group. This indicates that the combined treatment has a significant advantage in reducing adverse reactions. Both the ATP-IR bio-effect device and microwave therapy are noninvasive treatment methods that do not cause trauma to the patient’s body, thereby reducing adverse reactions caused by treatment.^[18,19]^ In addition, the non-invasiveness of the combined treatment also makes the treatment process more comfortable for patients and improves treatment compliance.

### 4.3. Clinical significance

The results of this study have important clinical significance. LS is a chronic disease that seriously affects the quality of life of patients. Although traditional glucocorticoid therapy can relieve symptoms to a certain extent, long-term use may bring a series of adverse reactions, such as skin atrophy and pigmentation. ATP combined with microwave therapy, as a noninvasive and painless physical treatment, can not only effectively relieve symptoms but also significantly reduce the occurrence of adverse reactions.^[20]^ This provides a new option for clinical treatment, especially when patients have poor tolerance to traditional treatment methods. Moreover, the long-term effect of the combined treatment is significant, indicating that it may also have potential advantages in preventing disease recurrence.^[21]^ This study followed up the cured, marked-effect, and effective cases for 1 year, and the results showed that the recurrence rate of the combined group was significantly lower than that of the other 3 groups (*P* <.05). This indicates that the combined treatment can not only effectively relieve symptoms but also reduce disease recurrence and improve patients’ long-term quality of life. Future research can further explore the effect of combined treatment in preventing recurrence and its applicability in different patient populations.

### 4.4. Limitations of the study

Despite the significant positive results obtained in this study, there are still some limitations.^[22–25]^ First, the sample size is relatively small, which may limit the generalizability of the results. Future research can increase the sample size to further verify the efficacy and safety of the combined treatment. Second, this study is a single-center study, which may have certain selection bias. Future research can adopt a multicenter, randomized controlled trial design to improve the reliability of the results. Finally, this study only followed up for 1 year, and the assessment of long-term effects may not be sufficient. Future research can extend the follow-up time to comprehensively evaluate the long-term effects of the combined treatment.

### 4.5. Future research directions

This study provides preliminary clinical evidence for the treatment of LS with ATP combined with microwave therapy, but further research is needed in the future to optimize the treatment plan and explore its mechanism of action.^[26]^ First, future research can explore the impact of different treatment parameters (such as the power of ATP and microwave, treatment time, treatment frequency, etc) on efficacy to determine the best treatment plan. Second, molecular biology techniques can be used to study the effects of ATP combined with microwave therapy on cytokines, inflammatory mediators, cell proliferation, and apoptosis in the skin tissue of patients with LS, to reveal its mechanism of action. In addition, long-term follow-up studies can be carried out to evaluate the long - term efficacy and safety of the combined treatment, as well as its long-term impact on patients’ quality of life.

## 5. Conclusion

In summary, ATP combined with microwave therapy has shown significant efficacy and safety in the treatment of LS in women. Its total effective rate and symptom relief effects are better than traditional glucocorticoid therapy, and the incidence of adverse reactions is significantly reduced. This result provides a new option for clinical treatment and has important clinical application value. Future research can further explore the long-term effects and potential for preventing recurrence of the combined treatment to optimize the treatment plan and improve patients’ long-term quality of life.

## Author contributions

**Conceptualization:** Xiaojiao Pei, Mengke Wang, Huan Wang, Yuling Jia.

**Data curation:** Xiaojiao Pei, Mengke Wang, Huan Wang, Yuling Jia.

**Formal analysis:** Xiaojiao Pei, Mengke Wang, Dan Lu, Huan Wang, Yuling Jia.

**Funding acquisition:** Dan Lu, Huan Wang, Yuling Jia.

**Investigation:** Dan Lu, Huan Wang, Yuling Jia.

**Writing – original draft:** Xiaojiao Pei, Mengke Wang, Yuling Jia.

**Writing – review & editing:** Xiaojiao Pei, Mengke Wang, Dan Lu, Yuling Jia.

## Correction

This article was originally published with an incorrect funding number has now been updated online from “No. 23146023” to “No. 231460233.”
